
JOURNAL INFORMATION
==============================
NLM Title Abbreviation: Wirtschaftsdienst
Journal ID: Wirtschaftsdienst
NLM Journal ID: 101086612

Wirtschaftsdienst (Hamburg, Germany : 1949)

ISSN: 0043-6275

ARTICLE INFORMATION
==============================
PMCID: PMC7765697
PMID: 33390618
DOI: 10.1007/s10273-020-2790-4
Article ID: 2790
Article version: 1
Subjects: Leitartikel

Lehren aus der Corona-Krise

Lessons from the Corona crisis

Erbe Susanne s.erbe@zbw.eu

Redaktion Wirtschaftsdienst, ZBW - Leibniz-Informationszentrum Wirtschaft, Neuer Jungfernstieg 21, 20354 Hamburg, Deutschland
Electronic publication date: 2020 Dec 26
Print publication date: 2020
Volume: 100
Issue: 12
First page: 902
Last page: 903
Copyright: © Der/die Autor(en) 2020
License: Dieser Artikel wird unter der Creative Commons Namensnennung 4.0 International Lizenz (https://creativecommons.org/licenses/by/4.0/deed.de) veröffentlicht.
Open Access wird durch die ZBW — Leibniz-Informationszentrum Wirtschaft gefördert.
License URL: https://creativecommons.org/licenses/by/4.0/

issue-copyright-statement: © The Author(s) 2020

==============================

Demnächst wird ein Jahr zu Ende gehen, das in vielfacher Hinsicht außergewöhnlich ist: für die Wirtschaft, die Politik, die Wissenschaft und das menschliche Miteinander. Das Coronavirus hat unser Leben verändert. Es traf die Menschheit global, und es traf sie unvorbereitet. Das zeigte sich unter anderem in der ersten Welle im März 2020, als die europäischen Staaten verzweifelt versuchten, Schutzausrüstung zu beschaffen – unkoordiniert und in Konkurrenz zueinander. Die Situation fiel eindeutig in die Kategorie Schwarzer Schwan, verbunden mit der größtmöglichen Unsicherheit. Um die Lage nicht außer Kontrolle geraten zu lassen, musste gehandelt werden, und zwar schnell. Dies ist die Stunde für „gutes Regieren". Tatsächlich hat aber ein autokratisches System wie China mit am erfolgreichsten auf die Krise reagiert. Infektionen werden vermieden, wenn soziale Begegnungen eingeschränkt werden. Dafür sind tiefe Eingriffe in die Grundrechte erforderlich. China hat drastische Lockdowns verhängt, die in demokratischen Gesellschaften kaum durchsetzbar wären. In Demokratien benötigt die Regierung hingegen das Vertrauen der Bevölkerung. Die Verantwortlichen müssen den Ernst der Lage transparent und verständlich vermitteln und möglichst evidenzbasiert handeln.

Am 12. März 2020 ordnete die Bundesregierung weitreichende Einschränkungen für den Einzelnen, aber auch für das Wirtschaftsleben an. Da das Virus sich global ausgebreitet hatte und Grenzen geschlossen wurden, kam es zu enormen Exportrückgängen. Lieferketten brachen zusammen – für ein international stark verflochtenes Land wie Deutschland eine ökonomische Bedrohung. Die Wirtschaftsforschungsinstitute schätzen in ihrem gemeinsamen Herbstgutachten, dass das Bruttoinlandsprodukt (BIP) in Deutschland 2020 um 5,4 % schrumpfen wird. Im Frühjahr waren die Institute in ihrer Prognose noch etwas optimistischer: Sie erwarteten einen Rückgang um 4,2 %. Dass es nicht viel schlimmer kam, ist nicht nur den automatischen Stabilisatoren zu verdanken, sondern auch diskretionären konjunkturpolitischen Maßnahmen von beispiellosem Umfang. Einig sind sich die Institute, dass das Kurzarbeitergeld und direkte Finanzhilfen für Unternehmen effiziente Instrumente waren, während die zeitlich befristete Senkung der Mehrwertsteuer unterschiedlich bewertet wurde. Über eine Aufstockung der Finanzmittel für Krisenverlierer*innen wie besonders stark betroffene Geringverdiener*innen aus dem Dienstleistungsbereich oder Soloselbständige wird sicher noch mehr diskutiert werden. Direkte Staatsbeteiligungen sind aber bereits beschlossen. Um den Zusammenbruch größerer Unternehmen zu verhindern, wurde das Engagement des Staats nicht nur akzeptiert, sondern sogar gewünscht: Für einen Wirtschaftsstabilisierungsfonds sind Mittel von insgesamt 600 Mrd. Euro vorgesehen, davon 100 Mrd. Euro für die Rekapitalisierung von Unternehmen. Und es gibt schon Interessenbekundungen von rund 60 Unternehmen. Der Staat als Retter! In diesem Umfang hätte man das vor einem Jahr noch nicht für möglich gehalten.

Wenig überraschend kann in einer solchen Situation die Schwarze Null nicht gehalten werden: Für 2020 rechnet die Gemeinschaftsdiagnose mit einem Rekorddefizit im Staatshaushalt von 183 Mrd. Euro. Damit könnte die Defizitquote auf 5,5 % in Relation zum BIP steigen, die Schuldenstandsquote auf ca. 70 %. Begleitet wird diese Finanzpolitik von der sehr lockeren Geldpolitik der EZB, die im Rahmen des Pandemic Emergency Purchase Programme (PEPP) bis Mitte 2021 Wertpapiere, vor allem Staatsanleihen, im Umfang von 1,35 Billionen Euro aufkaufen will. Begünstigt sind alle Staaten des

Euroraums. Vertrauenschaffende Maßnahmen und Risikosenkung in den anderen EU-Mitgliedstaaten nützen dem Nettoexporteur Deutschland. Zu all diesen Maßnahmen waren die Warnungen vor der Corona-Krise noch lauter: Inflation und Staatsbankrotte drohten, Zombie-Unternehmen würden gestützt, zukünftige Generationen hätten die Zeche zu zahlen, Staatsunternehmen arbeiteten ineffizient und der Wettbewerb werde beeinträchtigt. Aber unter Ökonom*innen ist eine Art Not-Keynesianismus ausgebrochen. Allgemein wird anerkannt, dass die Regierung schnell und entschieden handeln muss, dass große Summen investiert werden müssen, dass die Unsicherheit extrem groß bleibt und dass ein ganz wichtiges Ziel die Vertrauensbildung ist. Nach der ersten Erholung im Sommer und im Herbst mehren sich aber wieder die Stimmen, die eine Rückkehr zur Haushaltsdisziplin und weniger Staatsintervention fordern.

Auch in anderen Bereichen hat sich gezeigt, dass mehr geht als früher gedacht. Viele Arbeitnehmer*innen können im Homeoffice arbeiten und sparen sich damit den Arbeitsweg. An Besprechungen und Konferenzen nehmen viele nun digital teil. Für eine solche Neustrukturierung der Arbeitswelt, aber auch für eine Beschulung zuhause, ist eine umfassende, verlässliche und für jeden erreichbare digitale Infrastruktur sowie Digitalkompetenz erforderlich. Allerdings liegt Deutschland im europäischen Vergleich seit Jahren bei der Verbreitung der für Homeoffice nötigen breitbandigen Internetanschlüsse zurück. Für eine digitale Transformation sind noch erhebliche Investitionen in Infrastruktur und Köpfe nötig. Die Corona-Krise hat dazu beigetragen, dass diese Notwendigkeit allen bewusst geworden ist. Auch in der Klimapolitik erhoffen sich bestimmte Gruppen, dass es zu einem Wandel der Einstellungen kommt. Der Rückgang der Mobilität hat die $CO_2$-Emissionen deutlich sinken lassen: Wie eine Studie unter Mitarbeit von Hans Joachim Schellnhuber vom Potsdam-Institut für Klimafolgenforschung errechnet hat, sind die $CO_2$-Emissionen im Verkehr weltweit um 40 % gesunken (Januar bis Juni 2020 im Vergleich zu Januar bis Juni 2019). Den Klimaforschenden macht Mut, dass es gelungen ist, in so kurzer Zeit den Lebensstil und das Verkehrsverhalten zu ändern. Sie befürchten allerdings, dass dies nur von kurzer Dauer sein wird.

Die Corona-Krise hat zudem gezeigt, welche Macht dem Expertenurteil zukommt. Ohne Kenntnisse über die Letalität, die Infektiosität und die Ansteckungswege ist es für die Politik kaum möglich, zielgerichtet und effizient zu handeln. Virologie und Epidemiologie stellen daher nicht nur Medienstars, sondern auch unverzichtbare Berater*innen der Politik. Deren Einschätzungen haben sich im Laufe der Pandemie durchaus gewandelt. Hier konnten die Bürger*innen live beobachten, wie Wissenschaft funktioniert. Die Öffentlichkeit interessierte sich für die Aussagekraft empirischer Untersuchungen, für Repräsentativität und auch dafür, dass unterschiedliche empirische Verfahren unterschiedliche Ergebnisse hervorbringen können. Allerdings fehlt es noch immer an experimentellen Untersuchungen dazu, welche Maßnahmen wie wirken. Es scheint immer noch keine gesicherte Evidenz darüber vorzuliegen, wer sich wo ansteckt und welchen Beitrag geschlossene Kinos, Restaurants sowie Kitas und Schulen zu einer Verbesserung der Situation leisten. Dieses Wissen hätte seit Beginn der Epidemie unter Hochdruck gesammelt und verdichtet werden müssen, um für die zweite Welle gerüstet zu sein.

Die Krise ist noch nicht vorbei. Vielleicht entschärft im nächsten Jahr ein Impfstoff die Situation. Recht sicher kommen noch ein paar harte Wintermonate mit möglichen weiteren Einschränkungen. Die wirtschaftswissenschaftliche Forschung war in dieser Pandemie eng in das politische Handeln eingebunden und auch die Wissenschaftskommunikation hat in der Bevölkerung für Vertrauen gesorgt. Die Regierung hat wirtschafts- und finanzpolitisch schnell reagiert. Dieser pragmatische Ansatz, auch über Ideologiegrenzen hinweg, könnte eine Blaupause für die wirtschaftswissenschaftliche Beratung der Zukunft sein. Die evidenzbasierte ökonomische Beratung ist auch nach der Krise von großer Bedeutung für eine zielgerichtete Politikgestaltung.

Susanne Erbe
Redaktion Wirtschaftsdienst
s.erbe@zbw.eu

Susanne Erbe, Dipl.-Volkswirtin, ist stellvertretende Chefredakteurin des Wirtschaftsdienst.

